# Direct Dispensation of Prenatal Supplements With Iron and Anemia Among Pregnant People

**DOI:** 10.1001/jamanetworkopen.2023.32100

**Published:** 2023-09-01

**Authors:** Lisa R. Thiele, Elaine L. Duryea, Alexandra S. Ragsdale, Carrie A. Berge, Donald D. McIntire, David B. Nelson, Catherine Y. Spong

**Affiliations:** 1 Department of Obstetrics & Gynecology, University of Texas Southwestern Medical Center, Dallas, Texas; 2 Parkland Health, Dallas, Texas

## Abstract

**Importance:**

Postpartum transfusion is the most common indicator of severe maternal morbidity in the US. Higher rates of anemia are associated with a higher blood transfusion rate.

**Objective:**

To determine if providing, rather than recommending, supplements with iron at prenatal visits in a medically underserved community is associated with improved hematologic indices and reduced blood transfusion.

**Design, Setting, and Participants:**

In this quality improvement study, patients who delivered between May 13 and December 13, 2020, and thus were provided a prenatal supplement with iron throughout pregnancy were compared with those who delivered between January 1 and August 1, 2019, before supplements were dispensed. The study was conducted at Parkland Health, a safety net hospital in Dallas, Texas, with a 95% Medicaid-funded or self-pay population and included all patients who delivered at our institution during the study period with available hematologic data.

**Exposures:**

In the earlier cohort, all patients were recommended to obtain and take iron supplements. In the later cohort, prenatal supplements with iron were dispensed via clinic pharmacy to all patients during prenatal visits.

**Main Outcomes and Measures:**

Maternal hematocrit levels (28-32 weeks, delivery admission, and discharge), rates of anemia (hematocrit <30%), and postpartum transfusion for acute blood loss anemia were compared using χ^2^ and analysis of variance methods with *P* < .05 considered significant. The analysis took place in July of 2022.

**Results:**

Overall, 13 910 patients (98%) met inclusion criteria (mean age [SD], 27.9 [6.5] and 27.6 [6.5] years, mean [SD] body mass index at first visit, 29.2 [6.6] and 29.3 [6.6]). Mosty of the patients in both cohorts were of Hispanic ethnicity (76%). Providing iron-containing prenatal supplements was associated with higher average hematocrit levels at all time points including a mean difference of 1.27% (95% CI, 1.13%-1.42%) on admission for delivery, when compared with those who were not directly dispensed iron. Among patients prior to providing supplements, 18% had anemia on admission compared with 11% with iron-containing supplements dispensed (risk ratio [RR], 0.61; 95% CI, 0.56-0.66). Postpartum transfusion for acute blood loss anemia was reduced by one-third in patients after program implementation from 10 per 1000 to 6.6 per 1000 (RR, 0.62; 95% CI, 0.43-0.91).

**Conclusions and Relevance:**

In this quality improvement study, providing supplements with iron to patients at prenatal visits was associated with improved hematocrit levels, rates of anemia, and reduced transfusions unrelated to obstetric catastrophes among a predominantly Medicaid population.

## Key Points

**Question:**

Is directly dispensing a prenatal supplement with iron associated with anemia rates and transfusion among a predominantly Medicaid population?

**Findings:**

In this cohort study of more than 13 000 patients, directly dispensing iron supplements to patients was associated with improved hematocrit levels and reduced anemia rates compared with recommendation alone.

**Meaning:**

Directly providing prenatal iron supplements was significantly associated with improved average hematocrit levels throughout pregnancy and a reduction anemia rate throughout pregnancy and immediately postpartum.

## Teaser

This cohort study examines if providing, rather than recommending, supplements with iron at prenatal visits in a medically underserved community was associated with improved hematologic indices and reduced blood transfusion.

## Introduction

Iron supplementation during pregnancy has been well characterized to reduce maternal anemia. During pregnancy, maternal plasma volume expands by 40% to 50% and maternal erythrocyte volume increases by 15% to 20% to compensate for the increased metabolic demand of the expanding uterus and growing fetus.^1^ Increased iron demands during pregnancy and erythrocyte dilution due to plasma expansion commonly lead to anemia in the antepartum period with a prevalence as high as 27% among US populations.^1,2^ Anemia in pregnancy has been associated with higher rates of blood transfusion, postpartum anemia, and neonatal adverse outcomes including fetal growth restriction and perinatal mortality.^1,3^ Symptomatic anemia at the time of delivery may require blood transfusion postpartum, which is the leading cause of severe maternal morbidity (SMM) in the US.^3,4^ Transfusion carries risks of infection, transfusion reaction, and alloimmunization; and finite blood bank resources exist.^5,6^ Blood administration during obstetric catastrophe for acute hypovolemia and hemorrhage is difficult to prevent with prenatal iron supplementation; however, transfusion for symptomatic anemia in patients with preexisting anemia who experience normal blood loss at delivery may be preventable with preemptive repletion of iron stores.

Prenatal iron supplementation has been proven to reduce the rate of maternal anemia by up to 70%.^7^ The Centers for Disease Control and American College of Obstetrics and Gynecologists recommend starting low-dose iron supplementation, with at least 27 mg of elemental iron, at the first prenatal visit irrespective of hematologic status.^8,9,10^ This recommendation was based on the physiologic demands of pregnancy as well as low-risk profile of iron supplementation in pregnancy. Despite these recommendations, patients with low socioeconomic status may experience limited access to iron supplementation due to cost of supplements, access to transportation to access pharmacies or drug stores, and numerous other factors. Thus, iron deficiency anemia during pregnancy and postpartum is higher among patients in minority groups and patients from economically disadvantaged backgrounds.^9^ Although universal iron supplementation in pregnancy has been shown to reduce anemia, effective strategies to overcome barriers to medication and supplement access are less clear. We aimed to improve access to iron supplements, and thereby reduce anemia rates among our obstetric population, by reducing barriers to access including cost of supplements, access to transportation, and other factors. This study sought to evaluate the association of directly dispensing iron supplements during clinic visits to pregnant patients from a medically underserved community with improved hematologic indices, reduced maternal anemia, and transfusion for acute blood loss anemia at delivery.

## Methods

Parkland Health is a safety-net hospital located in Dallas, Texas, which predominantly provides care to patients with lower socioeconomic resources. Historically patients receiving prenatal care at Parkland Health were directly dispensed both iron and folic acid supplements. In 2015, due to reclassification of iron as nutritional supplement, rather than medication, this practice was limited to folic acid and patients were instructed to purchase iron supplements over the counter. A quality improvement analysis was performed in 2018, which revealed a higher level of anemia and blood transfusion at delivery in the latter cohort.

Thus, a public health initiative to improve access to prenatal iron supplements for Parkland patients was implemented starting September 25, 2019. All pregnant patients who presented for prenatal care at Parkland Health associated clinics were directly dispensed iron-containing prenatal supplements via a class-D pharmacy at their first prenatal visit and were offered refills at each subsequent appointment. Patients were provided with iron supplements throughout pregnancy and in the immediate postpartum period. During prenatal visits, clinicians counseled patients on the importance of taking prenatal vitamins with iron and then entered an order for the iron-containing prenatal supplements. Prior to leaving the clinic, nursing staff directly handed a prepackaged 90-capsule bottle of prenatal vitamins to patients. Funding for this study was provided by Parkland Health.

The prenatal supplement provided at Parkland Health clinics is a prescription iron-containing vitamin supplement designed for patients in the prenatal and postpartum period to improve nutritional status. The supplement is given as once daily dosing and contains 125 mg of iron from ferrous fumarate and polysaccharide iron complex, 1000 µg of folic acid, 40 mg of vitamin C, and 3 mg of niacin. This supplement was chosen for its once daily dosing and because they could manufacture a sufficient volume of supplements to meet the high demand at our institution. Parkland Health pharmacy purchased these prenatal supplements to be stocked in each of the women’s health clinics.

To assess the results of this initiative, we conducted a quality improvement study of all patients who underwent delivery at Parkland Health during 2 time points, between January 1 and August 1, 2019, (prior to supplement administration), and between May 13 and December 13, 2020, (when all patients would have had the opportunity to receive supplements with iron starting <10 weeks of gestation). Those in the first cohort (“pre–providing supplements”) were provided with a prenatal supplement that did not contain iron and counseled to purchase an additional over-the-counter iron supplement for daily use throughout their pregnancy. In the second cohort (“post–providing supplements”), patients were directly dispensed iron containing supplements during their first prenatal appointment via an in-clinic pharmacy, and then provided refills as needed. Intravenous iron infusion was not regularly available for patients with iron deficiency anemia at our institution during the duration of our study periods. Following the study period, a program of intravenous iron therapy for pregnant patients who do not respond to oral supplementation has been launched. This study was approved by the institutional review board of the University of Texas Southwestern Medical Center with site approval at Parkland Health. A waiver of informed consent was approved by the institutional review board for this study given the minimal risk to research participants. This report is written following the Standards for Quality Improvement Reporting Excellence (SQUIRE) 2.0 reporting guidelines for quality improvement research.

Maternal demographics, visit data, and perinatal outcomes of delivered patients were obtained from an obstetric quality database, which contains data extracted from the electronic medical record by dedicated research nurses according to standard protocols and definitions contained in a manual of operations, and routinely validated with cross-checks. All deliveries at Parkland Health during the defined study periods of infants larger than than 500 g, whether live or stillborn, were included. Laboratory data were extracted from the electronic health record and matched to the existing quality database. Data examined included race, parity, body mass index (BMI), age, gestational age at enrollment to prenatal care, incidence of postpartum hemorrhage, and transfusion requirement. Postpartum hemorrhage was defined as estimated blood loss greater than 500 cc in a vaginal delivery or greater than 1000 cc in a cesarean delivery consistent with institutional protocol. Maternal anemia was defined as a hematocrit less than 30%^11^ based on data from our patient population supporting this is less than the fifth percentile, and is consistent with national guidelines.^11^ Transfusion data included time from delivery to blood administration and number of units transfused.

Transfusion for acute blood loss anemia was defined as transfusion of 1 to 2 units of packed red blood cells or whole blood counts postpartum in the admission for delivery. The electronic medical records of patients who required transfusion were reviewed for symptoms at time of transfusion. Transfusion data for a patient was excluded if the transfusion was performed secondary to hypovolemia in the setting of obstetric catastrophe including abruption, previa, atony, operative complication, and vaginal trauma, or symptomatic hypovolemia including oliguria and hypotension.

### Primary and Secondary Outcomes

The primary outcome was the average hematocrit values at the measured time points (24-32 weeks, admission for delivery, prior to discharge, and at postpartum follow-up). Hematocrit level was chosen because it is the standard measurement at our institution for anemia in the prenatal and postpartum period. There were 2 secondary outcomes. First, the measure of hematocrit was used to determine if rate of anemia in the population, defined as proportion of patients with a hematocrit level lower than 30% per previous studies at our institution, changed with universal iron supplementation.^11^ Second, we compared incidence of transfusion for symptomatic blood loss anemia in the immediate postpartum period not related to obstetric catastrophe between the 2 cohorts.

### Statistical Analysis

Primary analysis was performed to examine maternal hematocrit levels in the third trimester, at admission for delivery, and postpartum prior to hospital discharge. Anemia rates and rates of transfusion for symptomatic blood loss anemia were compared. Statistical analysis included χ^2^ and analysis of variance. Adjusted analysis for age, race, BMI, and nulliparity was completed using log-binomial regression through a generalized linear model with a log link function and binomial distribution for changes in anemia rate. The Osius and Rojek and Stukel statistics were used to examine goodness of fit for the regression of categorical variables. Analysis of covariance was used to complete adjusted analysis for age, race and ethnicity, BMI, and nulliparity for changes in postpartum transfusion. Results at *P* < .05 were considered significant. Analysis was conducted using SAS statistical software (version 9.4; SAS Institute, Inc).

## Results

**Figure 1.**
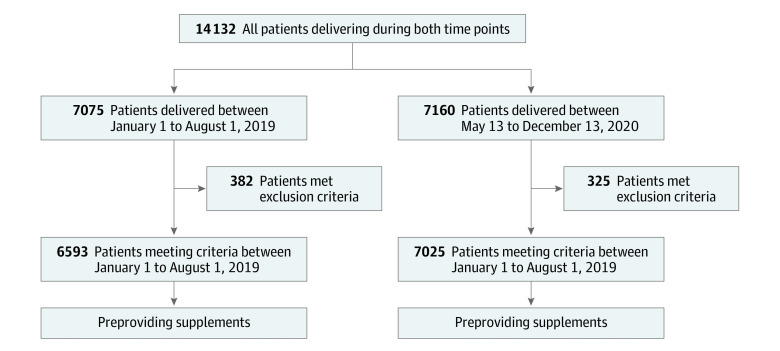
Flow Diagram of Study Participants Included

There were 7075 patients delivered between January 1 and August 1, 2019, and 7160 between May 13 and December 13, 2020; most patients in both cohorts were multiparous, overweight (mean [SD] BMI on enrollment to prenatal care 29.2 [6.6] and 29.3 [6.6]), and of Hispanic ethnicity (76% in both cohorts) (Table 1). Of the 14 235 total patients delivered during the study period, 13 910 (98%) met inclusion criteria of delivering an infant over 500 g at our institution (Figure 1). The cohorts were comparable in terms of age, race and ethnicity, insurance payer status, and parity. The median (first quartile, third quartile) gestational age at enrollment to prenatal care was 12 (9, 19) weeks in the recommended supplements cohort and 11 (9, 17) weeks in the provided supplements cohort which represented a statistically significant but likely not clinically significant difference in the 2 cohorts.

**Table 1.** Demographics of Participants

Characteristic	Patients, No. (%)		*P* value
	Pre–providing supplements (January 1 to August 1, 2019), No. (%)	Post–providing supplements (May 13 to December 13, 2020), No. (%)	
Patients delivered, No.	6886	7025	
Age, mean (SD), y	27.9 (6.5)	27.6 (6.5)	.02
Non-Hispanic Black race	1136 (16)	1223 (17)	.15
Hispanic ethnicity	5263 (76)	5327 (76)	.40
Nulliparity	2023 (29)	2078 (30)	.79
BMI ≥40	433 (6)	471 (7)	.32

Abbreviation: BMI, body mass index (calculated as weight in kilograms divided by height in meters squared).

No difference in the rates of postpartum hemorrhage (12% and 11%, *P* = .08), HELLP syndrome (0.3% and 0.5%, *P* = .13), and placental invasion (2 patients pre–providing supplements and 4 patients post–providing supplements, *P* = .43) were found. Antepartum transfusion was rare in both cohorts with 19 patients in the earlier cohort and 10 patients in the later cohort requiring transfusion in pregnancy prior to delivery admission. A total of 20 386 bottles of the prenatal supplement with iron were distributed to patients between May 13 and December 13, 2020, consistent with dispensation of 260 capsules, or 2 to 3 refills, to each patient.

**Figure 2.**
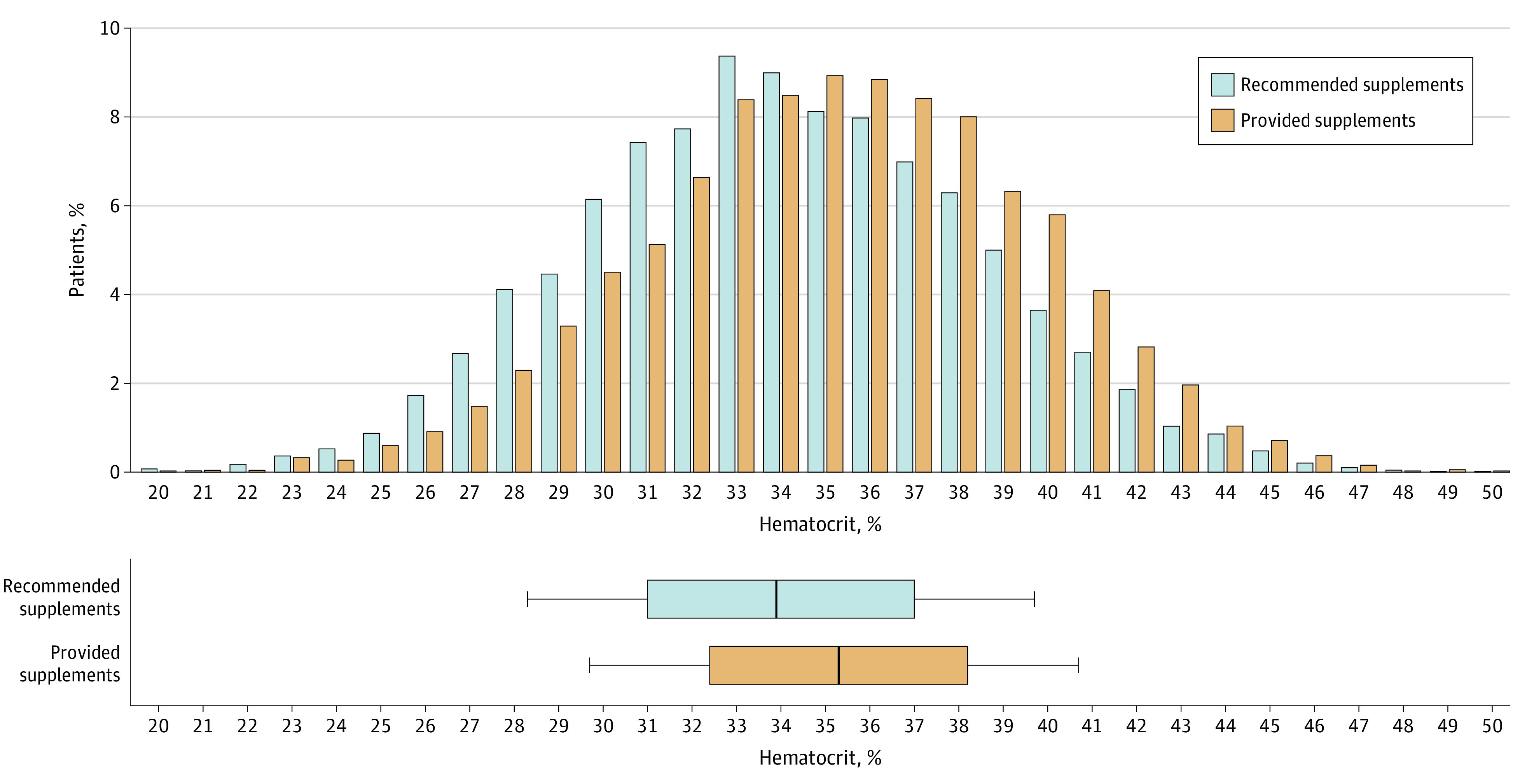
Hematocrit Levels on Admission For Delivery The boxes show the mean (SD) hematocrit levels and the whiskers represent the 95% CIs. Patients in the post–providing supplements cohort had higher hematocrit levels when presenting for delivery compared with patients who were recommended, not provided, iron supplements.

The second cohort, which was provided prenatal vitamin supplements with iron, had consistently higher hematocrit levels in the third trimester, on admission for delivery, prior to discharge postpartum, and at the postpartum follow-up clinic visit (Table 1). The mean (SD) third trimester hematocrit level prior to iron supplement distribution was 34.7% (3.2%), which improved to 35.2% (3.1%) among those provided with supplements (mean difference, 0.45%; 95% CI, 0.33%-0.51%). The cohort which was recommended supplements had a mean (SD) hematocrit level of 34.0% (4.4%) on admission for delivery, this was significantly lower than the cohort that was provided supplements with a mean (SD) hematocrit level of 35.3% (4.3%) (mean difference, 1.27%; 95% CI, 1.13%-1.42%). This reflected an overall shift in hematocrit levels toward higher values in the provided supplements cohort with a reduced number of patients presenting for delivery with low and very low hematocrit levels after being provided with prenatal supplements with iron (Figure 2). Mean (SD) hemoglobin levels on admission for delivery were higher among the provided supplements cohort (11.6 [1.6] g/L) compared with the recommended supplements cohort (11.1 [1.7] g/L). Similarly, the cohort that received iron-containing supplements had significantly higher hematocrit levels prior to discharge with a mean (SD) of 31.1% (3.7%) compared with 30.8% (3.7%) (mean difference, 0.36%; 95% CI, 0.23%-0.48%) in the earlier cohort. At the postpartum visit an average of 20 days after discharge from delivery admission, the recommended supplements cohort had a mean (SD) hematocrit level of 38.8% (3.6%) which was significantly lower than the cohort provided with supplements with a mean (SD) hematocrit level of 39.1% (3.7%) (mean difference, 0.29%; 95% CI, 0.15%-0.43%) (Table 2).

**Table 2.** Clinical Outcome Data

Outcome	Patients, No. (%)		Adjusted RR (95% CI)^a^
	Pre–providing supplements (January 1 to August 1, 2019)	Post–providing supplements (May 13 to December 13, 2020)	
Postpartum transfusion, No. (No. per 1000)^b^	71 (10.0)	46 (6.6)	0.62 (0.43-0.91)
Hematocrit 24-32 weeks, mean (SD)	34.7 (3.3)	35.2 (3.1)	0.45 (0.33-0.51)^c^
Anemia 24-32 weeks	377 (7)	278 (5)	0.68 (0.59-0.79)
Hematocrit at delivery admission, mean (SD)	34.0 (4.4)	35.3 (4.3)	1.27 (1.13-1.42)^c^
Anemia delivery admission	1237 (18)	782 (11)	0.61 (0.56-0.66)
Hematocrit at delivery discharge, mean (SD)	30.8 (3.7)	31.1 (3.7)	0.36 (0.23-0.48)^c^
Anemia delivery discharge	2810 (41)	2550 (36)	0.89 (0.85-0.93)

Abbreviation: RR, risk ratio.

^a^ Adjusted for: age, race and ethnicity, body mass index, insurance payer status, and nulliparity through logistic regression.

^b^ Transfusion for acute blood loss anemia included those transfused 1 to 2 units of packed red blood cells or whole blood and excluded transfusions for abruption, previa, atony, operative complication, vaginal trauma, sickle cell, hypovolemia, oliguria or the receipt of platelets, plasma, or cryoprecipitate.

^c^ Adjusted mean difference (95% CI) evaluated at the midpoint of adjustment factors.

**Figure 3.**
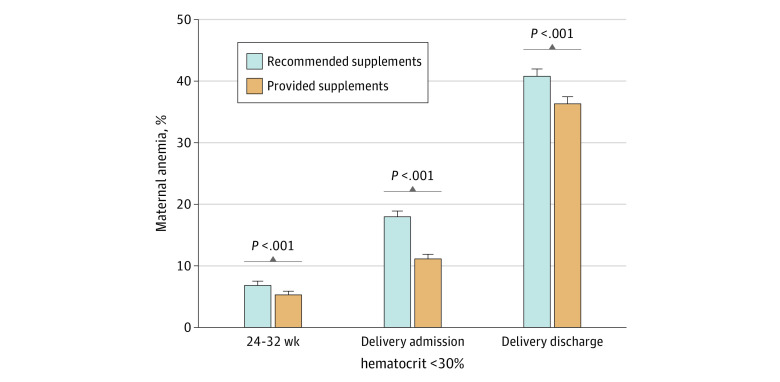
Rates of Anemia During Pregnancy, at Delivery Admission, and at Discharge Before and After Dispensing Supplements With Iron

Similarly, the rate of maternal anemia was significantly lower among patients provided with iron-containing supplements in the antepartum and immediate postpartum period (Figure 3). The prevalence of anemia among the recommended supplements cohort in the third trimester was significantly higher than the provided supplements cohort at the same time point (risk ratio [RR], 0.68; 95% CI, 0.59-0.79). On admission, the prevalence of maternal anemia was 7% lower in the provided supplements cohort compared with recommended supplements (RR, 0.61; 95% CI, 0.56-0.66). Prior to discharge, the cohort that was recommended iron supplements had a prevalence of maternal anemia of 41%, whereas the cohort that received prenatal vitamins with iron had a prevalence of 36% (RR, 0.89; 95% CI, 0.85-0.93). Results remained significant when adjusted analysis using logistic regression was performed for demographic factors. No significant lack of fit was observed when using the Osius and Rojek and Stukel statistics.

When comparing transfusion rates for acute blood loss anemia not due to obstetric catastrophe or hypovolemia, those directly receiving iron were less likely to require transfusion. Transfusion for acute blood loss anemia not related to obstetric catastrophe occurred in 71 (1.0%) of deliveries in the recommended supplements group compared with 46 (0.7%) in patients directly dispensed prenatal supplements with iron (RR, 0.62; 95% CI, 0.43-0.91). Results remained significant when adjusted using analysis of covariance for demographic factors.

## Discussion

In this cohort study of 13 910 deliveries, we found that directly dispensing prenatal supplements with iron at prenatal visits was associated with higher average hematocrit levels, lower rates of antepartum and postpartum anemia, and a one-third reduction in blood transfusion for symptomatic anemia. This novel strategy addresses a critical question of how to affect maternal morbidity and mortality, especially in an underserved population. Although prenatal iron is routinely recommended, these findings suggest that providing the supplement directly to the patient was associated with tangible clinical outcomes and a reduction in the most common cause of maternal morbidity.

This study provides data to support that preventative iron supplementation in pregnancy reduces maternal anemia. A meta-analysis by Pena-Rosas, et al^7^ found a 70% reduction in maternal anemia at term with preventative iron supplementation in pregnancy. This analysis defined anemia at a hemoglobin level of 11.0 g/L and saw an overall decrease in prevalence of anemia from 35.71% to 13.06% with high heterogeneity in the results. In the current study, we observed a change in prevalence of anemia from 18% to 11% on admission for delivery. Our study defined maternal anemia as hematocrit levels lower than 30% (as defined in our population)^11^ and measured hematocrit levels on admission. A 2018 study by Siekmans and colleagues^12^ surveyed patients across 7 different countries in Asia and Africa to identify barriers to prenatal iron and folic acid supplementation. Their findings demonstrated that systemic barriers including coverage of prenatal vitamins, distribution of supplements, and confusion over the appropriate supplements required in pregnancy all contributed to patient adherence. Our data support these findings in a medically underserved American population by demonstrating an association between improved hematologic indices on a population level and a reduction in barriers to accessing prenatal supplements through directly dispensing vitamins in clinic. These data also demonstrate improved outcomes when a program designed to reduce cost to the patient and increase convenience was implemented.

This study provides evidence that directly dispensing iron-containing prenatal vitamins to patients at prenatal visits was associated with improved hematologic outcomes and reduced transfusion for symptomatic acute blood loss anemia in the immediate postpartum period. Hospital systems that service a medically underserved and at-risk populations may now consider these data when designing similar programs or when assessing methods of reducing maternal morbidity due to blood transfusion.

### Strengths and Limitations

The population size and standardized, protocol driven care at 1 institution is a major strength of this study. By evaluating data from over 7000 patients in each cohort, we assessed the outcome of directly dispensing prenatal iron supplements on a population level. The study is potentially limited in its generalizability to other populations; however, previous studies completed at our institution did not demonstrate a change in hematologic indices at delivery when comparing 2 demographically unique populations.^13^

This study is also limited by the availability of indirect adherence data in both cohorts. Our clinics dispensed a total of 20 383 ninety-capsule bottles of the iron-containing prenatal supplement during the study period, which suggests that patients were requesting an average of 2 refills of the supplements at prenatal visits. The high number of dispensed bottles paired with the overall improvement in hematocrit levels and reduction in anemia prevalence suggests increased adherence among the later cohort when iron supplements were directly dispensed to patients. This study evaluated outcomes using a supplement with once daily dosing.

The availability of hematocrit levels alone is a potential limitation of our study. Hematocrit level was chosen as the primary outcome because it is regularly used in screening for hematologic status among obstetric patients at our institution, particularly at outpatient prenatal visits. Because hemoglobin levels are related to hematocrit levels, we presume that hemoglobin trends would have a similar result as hematocrit and this is demonstrated on admission for delivery, when complete blood counts are routinely ordered on all obstetric patients.

Another potential limitation of our study is the separation of the cohorts by the COVID-19 pandemic. It is possible that patient compliance in the post–providing supplements cohort was affected by increased time at home during the COVID-19 lockdown or increased health awareness during this time. Given that economic hardships related to the COVID-19 pandemic disproportionally affected patients of lower socioeconomic status,^14^ it could be assumed that nutritional deficiencies related to increased food insecurity and difficulty accessing supplements would increase among our population. Despite this, we found that directly dispensing iron supplements free of charge to patients was associated with an improvement in anemia rates.

## Conclusions

This study found improvement in hematologic indices and reduction in transfusion for acute blood loss anemia not associated with obstetric catastrophe following implementation of a public health initiative to directly provide pregnant patients with iron supplements. These data suggest that adherence may improve when supplements are directly provided to patients at their regular prenatal visits. Given this evidence, hospital systems should consider implementing programs to improve access to iron-containing prenatal vitamins, particularly when serving a medically at-risk population.

## Notes

Supplement.
Data Sharing Statement
