# Error in Figure 1

**DOI:** 10.1001/jamanetworkopen.2023.38311

**Published:** 2023-09-28

In the Original Investigation titled “Direct Dispensation of Prenatal Supplements With Iron and Anemia Among Pregnant People,”^1^ published on September 1, 2023, there was an error in Figure 1. The final boxes now correctly read “preproviding supplements” and “postproviding supplements.”
